# Blockage of NOX2/MAPK/NF-*κ*B Pathway Protects Photoreceptors against Glucose Deprivation-Induced Cell Death

**DOI:** 10.1155/2017/5093473

**Published:** 2017-09-11

**Authors:** Bin Fan, Bei-Fen Wang, Lin Che, Ying-Jian Sun, Shu-Yan Liu, Guang-Yu Li

**Affiliations:** Department of Ophthalmology, Second Hospital of JiLin University, Changchun 130041, China

## Abstract

Acute energy failure is one of the critical factors contributing to the pathogenic mechanisms of retinal ischemia. Our previous study demonstrated that glucose deprivation can lead to a caspase-dependent cell death of photoreceptors. The aim of this study was to decipher the upstream signal pathway in glucose deprivation- (GD-) induced cell death. We mimicked acute energy failure by using glucose deprivation in photoreceptor cells (661W cells). GD-induced oxidative stress was evaluated by measuring ROS with the DCFH-DA assay and HO-1 expression by Western blot analysis. The activation of NOX2/MAPK/NF-*κ*B signal was assessed by Western blot and immunohistochemical assays. The roles of these signals in GD-induced cell death were measured by using their specific inhibitors. Inhibition of Rac-1 and NOX2 suppressed GD-induced oxidative stress and protected photoreceptors against GD-induced cell death. NOX2 was an upstream signal in the caspase-dependent cell death cascade, yet the downstream MAPK pathways were activated and blocking MAPK signals rescued 661W cells from GD-induced death. In addition, GD caused the activation of NF-*κ*B signal and inhibiting NF-*κ*B significantly protected 661W cells. These observations may provide insights for treating retinal ischemic diseases and protecting retinal neurons from ischemia-induced cell death.

## 1. Introduction

Acute energy failure is one of the critical factors contributing to the pathogenic mechanisms of retinal ischemia. Decreased glucose levels result in the death of photoreceptors and subsequent vision injury because over 80% of glucose uptake by photoreceptors is used for anaerobic glycolysis, producing more than half of the photoreceptor ATP when O_2_ is available [1]. When mitochondria fail to produce cell energy, programmed cell death may be initiated. Our previous study revealed that acute energy depletion or glucose deprivation (GD) induced a caspase-dependent death in photoreceptor cells, since the pan caspase inhibitor, Z-VAD-fmk, remarkably rescued cells from GD-induced cell death and the active form of caspase-3 was detected in GD-treated cells. In addition, mitochondria had lower mitochondrial-membrane potential (Δ*ψ*m) followed by the subsequent release of cytochrome C into the cytosol [2]. Although the main pathway of GD-induced programmed cell death has been clarified, the upstream signal that triggers caspase-dependent cell death is still unclear. In GD-treated cells, we found that acute energy depletion caused excessive intracellular reactive oxygen species (ROS) generation. Therefore, in this study we focused on oxidative stress injury and related responses caused by GD.

NAD(P)H oxidases (NOXes) catalyze superoxide production by transferring an electron from NADPH to molecular oxygen thus generating extracellular superoxide and its downstream ROS, which is regarded as a primary source for intracellular ROS [3]. There are five NOX family members (NOX1–5), and NOX2 is predominantly present in the brain and neurons. NOX2 consists of two membrane subunits, gp91^-phox^ and p22^-phox^, which form the catalytic core, and the cytosolic components, p67^-phox^, p47^-phox^, and p40^-phox^, and GTPase Rac [4]. The Rho-like GTPase, Rac1, is necessary for structural and functional activation of the NOX complex by ameliorating the gp91^phox^-p67^phox^ interaction [5].

Members of the MAPK (mitogen-activated protein kinase) family play a critical role in oxidative stress-induced neuronal death. In response to oxidative stress, the highly conserved serine/threonine kinases in the MAPK signaling cascades connect cell surface receptors to regulatory targets [6]. The MAPK signaling pathways mainly activate three kinase subfamilies: c-Jun NH2-terminal kinase (JNK), the p38 kinases, and the extracellular signal-regulated kinases (ERK) [7]. MAPKs are activated through upstream kinases, including mitogen-activated protein kinase kinase 1 and 2 (MKK1/2), MKK3/6, and MKK4/7, which can reversibly phosphorylate threonine and tyrosine residues of the TXY motif in the catalytic domain. ERK and p38 are in general activated by MKK1/2 and MKK3/6, respectively, whereas JNK is activated by MKK4/7 [8]. Once activated, MAPKs phosphorylate several cellular substrates to propagate the signaling cascades, thus leading to many forms of cellular responses, including proliferation, differentiation, or programmed cell death [9].

In this study, we mimicked acute energy depletion by using GD in retinal photoreceptor cells (661W) to further illustrate the role of NOX2 and MAPK family members in GD-induced cell death. In addition, since NF-*κ*B (nuclear factor kappa B), an important transcription regulator, was influenced by cellular redox status, its effect on acute energy depletion-damaged photoreceptor was investigated as well.

## 2. Materials and Methods

### 2.1. Chemicals and Reagents

Cell culture media and additives were from HyClone Company (Beijing, China). p-ERK, p-P38, p-JNK, NF-*κ*B, Bcl-2, and Bax antibodies were purchased from Beyotime Biotechnology (Shanghai, China). Caspases-3 antibody was from Cell Signaling Technology. HO-1 antibody was purchased from Bioworld Technology (Minnesota Minneapolis, US). Actin and gp91 antibodies were from Signalway Technology (St. Louis, MO). Phosphorylated IkB*α* and IkB*α* antibodies were purchased from Santa Cruz (Shanghai, China). DCFH-DA, JC-1, Hoechst, PI, PDTC, PD98059, SB203580, U0126, and SP60012 were purchased from Beyotime Biotechnology (Shanghai, China). Apocynin (APO), NSC23766 (NSC), and other reagents were purchased from Sigma-Aldrich (Shanghai, China).

### 2.2. Cell Culture

A 661W photoreceptor cell line was generously provided by Dr. Muayyad Al-Ubaidi (University of Oklahoma Health Sciences Center, Oklahoma, USA). 661W cells were cloned from retinal tumors from a transgenic mouse line and expressed simian virus 40T antigen under the control of the human interphotoreceptor retinol-binding protein promoter. 661W cells behave as photoreceptor cells and express blue and green cone pigments, transducin, and cone arrestin, but they do not express retinal pigment epithelial cell-specific proteins. Furthermore, 661W cells are sensitive to photooxidative stress, similar to normal retinal photoreceptor cells [10].

Cells were grown in Dulbecco's modified Eagle's medium supplemented with 10% heat-inactivated fetal calf serum and 1% penicillin/streptomycin, at 37°C in a humidified atmosphere with 5% CO_2_. For the GD experiment, the 661W cells were cultured in 96- or 24-well plates for 24 h in normal medium, washed three times with PBS, and then cultured in glucose-free medium at the indicated time points. For signals' study assay, all the inhibitors such as NOX2 inhibitor, Rac1 inhibitor, MAPK subfamilies' inhibitors, and NF-*κ*B inhibitor were applied at indicated concentrations during GD and cell viability/cell death percentage were determined.

### 2.3. Propidium Iodide/Hoechst Staining

After GD at the indicated time points, cells were stained with the staining solution (2 *μ*g/ml) and incubated in the dark for 10 min at room temperature. PI-positive cells were visualized under an inverted fluorescence microscope (Olympus, Japan). For cell death assessment, cells were harvested by trypsinization, rinsed with PBS, and scored using flow cytometry (FACSCanto II; BD Bioscience, CA). The cell death percentage was calculated as PI-positive cells/Hoechst stained cells times 100.

### 2.4. MTT Assay

Cells were seeded in 96-well plates (1 × 10^4^ cells/well). After treatment, cells in each well were incubated in 10 *μ*l MTT (5 mg/ml, Sigma) at a final concentration of 500 *μ*g/ml for 1 h at 37°C and then the supernatant was removed. Next, 100 *μ*l DMSO was added to each well and the plate was incubated at 37°C overnight. Absorbance (OD_570/650 nm_) was measured using an Infinite M200pro microplate reader (Tecan, Mannedorf, Switzerland).

### 2.5. Mitochondrial-Membrane Potential Assay (Δ*ψ*m)

Cells were cultured in 96-well plates in normal or glucose-free medium for the indicated times. Then, medium was removed, and cells were washed with Ca^2+^/Mg^2+^-free PBS. Cells were stained with JC-1 (10 *μ*g/ml) for 30 min at 37°C and were examined under a fluorescence microscope (Olympus, Japan). Using flow cytometry, JC-1-stained cells were quantified. After incubation with the JC-1 staining solution at 37°C in the cell incubator for 30 min, cells were washed with JC-1 staining buffer two times and then the percentage of positive JC-1 staining was analyzed with a flow cytometer (FACSCanto II; BD Bioscience, CA) using 488 nm excitations with 530/30 nm and 585/42 nm. The percentage of JC-1-positive staining was calculated as JC-1-positive cells/total cells times 100.

### 2.6. Intracellular ROS Measurement

Intracellular ROS was measured with an oxidation-sensitive fluorescence probe (DCFH-DA). Cells were cultured in 6-well plates in normal medium or glucose-free medium for 6, 16, and 24 h and washed twice with fresh medium, then incubated in 10 *μ*M DCFH-DA at 37°C for 20 min. Oxidized 2,7-dichlorofluorescein (DCF) fluorescence was visualized under a fluorescence microscope (Olympus, Japan). Fluorescence intensities were quantitatively analyzed using ImageJ software (NIH, Bethesda, MD).

### 2.7. Immunohistochemistry

Fixed cells were washed three times, and the cells were permeabilized and blocked for nonspecific epitopes with PBA buffer (1% BSA, 0.1% saponin, and 0.05% NaN_3_ in PBS) together with 2% donkey serum for 30 min at room temperature. Goat anti-NF-*κ*B primary antibody (Beyotime Biotechnology, Shanghai, China, 1 : 500) was incubated overnight in PBA at 4°C. After the cells were washed three times with PBA, they were incubated for 30 min in an Alexa-labeled secondary Ab (1 : 1000). Nuclei were counterstained with 1 *μ*g/ml Hoechst. Cell fluorescence was detected and captured using a fluorescence microscope (DM RA; Leica).

### 2.8. Western Blot Analysis

Cells (661W) were sonicated in protein lysate buffer and a bicinchoninic acid assay was used to estimate protein. An equal amount (20 *μ*g) of cell lysate was dissolved in sample buffer, and samples were boiled for 3 min. Electrophoresis was performed with 10% polyacrylamide gels containing 0.1% SDS. Proteins were transferred to nitrocellulose membranes, and blots were incubated for 3 h at room temperature with primary antibodies and then incubated with the appropriate biotinylated secondary antibodies. Signals were developed using enhanced chemiluminescence, and images were captured using a microscope equipped with a CCD camera (Tanon, Shanghai). Densitometric analysis was performed using Quantity One software (Bio-Rad Laboratories).

### 2.9. Statistical Analysis

Each experiment was repeated at least three times. Data are expressed as means ± SEM. Differences between means were evaluated using one-way ANOVA followed by a Bonferroni test (significance was set at *P* < 0.01).

## 3. Results

### 3.1. Inhibiting NOX2 Suppresses GD-Induced Oxidative Stress

**Figure 1:**
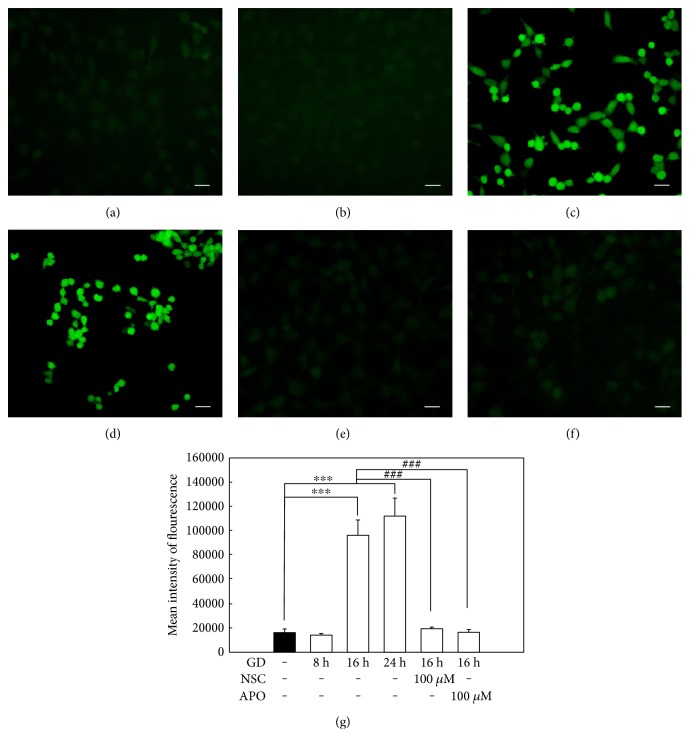
Inhibition of NOX2 suppresses GD-induced oxidative stress. (a)–(f) Intracellular ROS was measured with DCFH-DA identified by green fluorescence. (a)–(d) 661W cells were cultured in normal medium (a) or glucose-free medium for 8 h (b), 16 h (c), and 24 h (d). (e)-(f) 661W cells were treated with 100 *μ*M NSC (e) or 100 *μ*M APO (f) during the course of GD for 16 h. Scale bar = 25 *μ*m. (g) Fluorescence intensities were measured and relative fluorescence was statistically analyzed, scale bar = 25 *μ*m. The results are presented as means ± SEM (*n* = 6, ^∗∗∗^*P* < 0.01 compared to the control group, ^###^*P* < 0.01 compared to the vehicle group; one-way ANOVA and Bonferroni test).

**Figure 2:**
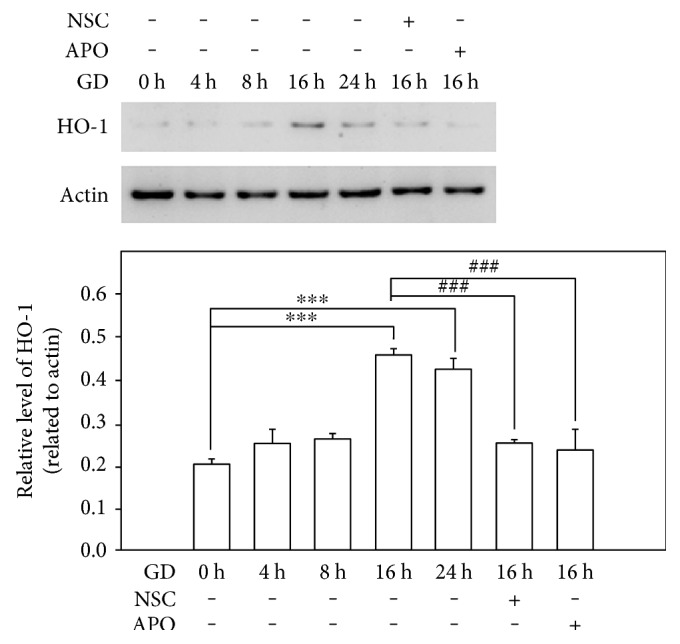
Inhibition of NOX2 suppresses HO-1 upregulation. 661W cells were cultured in normal, glucose-free medium, or in the presence of 100 *μ*M NSC or APO for indicated time periods. Cell lysates were collected and subjected to Western blot analysis of HO-1. Actin protein was used as a loading control. Protein bands on the Western blots were scanned, and the intensity was determined by optical density measurements. The results are presented as means ± SEM (*n* = 3, ^∗∗∗^*P* < 0.01 compared to the control group, ^###^*P* < 0.01 compared to the vehicle group; one-way ANOVA and Bonferroni test).

Glucose deprivation induced massive production of intracellular ROS in photoreceptors. As shown in Figure 1 (Figures 1(a), 1(b), 1(c), 1(d), and 1(g)), the cells cultured in glucose-free medium generated significantly increased levels of ROS at 16 h and 24 h when compared to the control group (*n* = 6, *P* < 0.01) evidenced by the robust green fluorescence staining of cells using the DHE assay. Next, since NOX2 normally functions as a primary source to generate ROS, we determined its influence on ROS suppression with a NOX2 inhibitor, APO, or a RAC1 inhibitor, NSC. Figure 1 (Figures 1(e), 1(f), and 1(g)) shows that treatment with 100 *μ*M NSC or 100 *μ*M APO at 16 h after GD remarkably reduced cellular ROS generation. This was evidenced by the significantly decreased green fluorescence when compared to the vehicle-treated group (*n* = 6, *P* < 0.01). Furthermore, heme oxygenase-1 (HO-1), an inducible and redox-regulated enzyme, is currently considered to have an important role in assessing cellular status during oxidative stress. We measured the effect of NOX2 inhibition on HO-1 expression with Western blot. Consistently, GD markedly upregulated HO-1 at 16 h and 24 h compared to the control group (*n* = 3, *P* < 0.01), while treatment with 100 *μ*M NSC or 100 *μ*M APO significantly suppressed the GD-induced expression of HO-1 at 16 h when compared to the vehicle group (*n* = 3, *P* < 0.01) (Figure 2). These results suggest that cells undergo severe oxidative stress during the course of GD, yet NOX2 signal plays a critical role in the GD-induced ROS generation, and blocking NOX2 could effectively suppress intracellular ROS generation.

### 3.2. Inhibiting NOX2 Protects Photoreceptors from GD-Induced Cell Death

**Figure 3:**
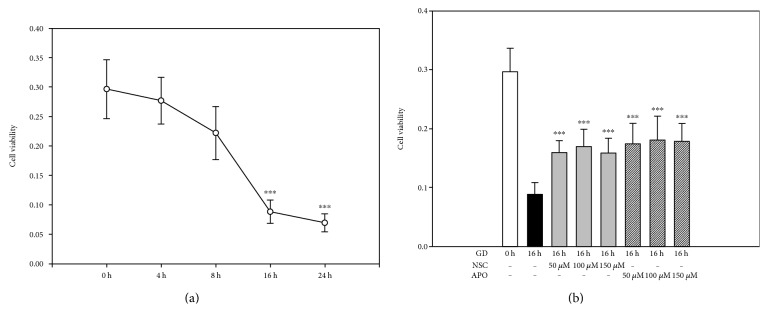
Inhibition of NOX2 increases viability of 661W cells. (a) 661W cells were cultured in glucose-free medium for indicated times, and cell viability was determined with the MTT assay. (b) 661W cells were cultured in glucose-free medium for 16 h in the presence of APO or NSC (50–150 *μ*M), and cell viability was determined with the MTT assay. The results are presented as means ± SEM (*n* = 3, ^∗∗∗^*P* < 0.01 compared to the vehicle group; one-way ANOVA and Bonferroni test).

**Figure 4:**
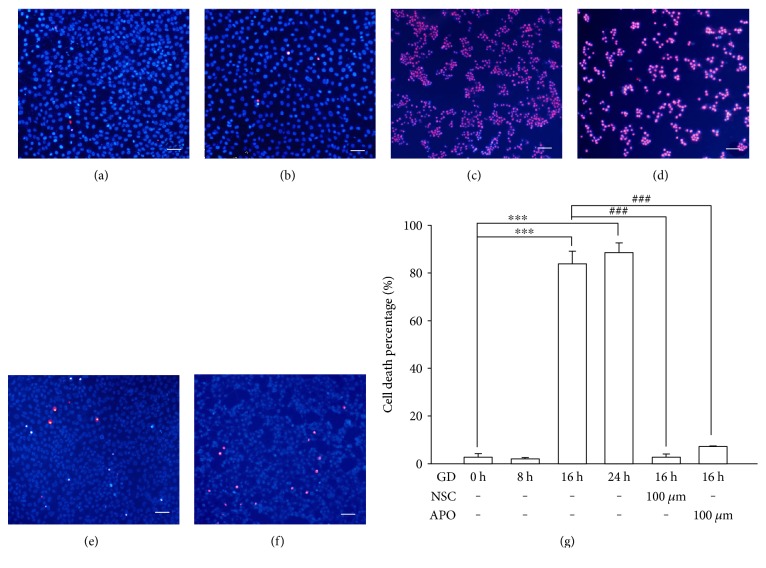
Inhibition of NOX2 reduces cell death percentage. Cell death was evaluated by PI/Hoechst staining. PI-positive cells were identified with purple fluorescence and total cells were counter stained with Hoechst showing blue fluorescent nucleus. (a)–(d) 661W cells were cultured in normal medium or glucose-free medium for 0 h, 8 h, 16 h, and 24 h. (e), (f) 661W cells were cultured in glucose-free medium for 16 h in the presence of 100 *μ*M NSC or APO. Scale bar = 200 *μ*m. (g) Quantitative assessment of cell death was performed with flow cytometry. The cell death percentage was calculated as PI-positive cells/Hoechst stained cells times 100. Assays were performed in quadruplicate, and data are shown as means ± SEM (^∗∗∗^*P* < 0.01 compared to the control group, ^###^*P* < 0.01 compared to the vehicle group; one-way ANOVA and Bonferroni test).

Glucose is the major source of energy supply for photoreceptors to maintain physiological activity, and thus GD is expected to cause severe damage in photoreceptors. As shown in Figure 3(a), the cells cultured in glucose-free medium had slightly decreased cell viability at 8 h, but a rapid decline in cell viability was detected with the MTT assay after 8 h and most cells were not viable at 16 h and 24 h when compared to the control group (*n* = 6, *P* < 0.01). However, the inhibition of NOX2 and Rac1 significantly attenuated GD-induced decrease in cell viability. As shown in Figure 3(b), treatment with NSC or APO at concentrations between 50 *μ*M and 150 *μ*M exhibited marked neuroprotective effects at 16 h after GD, which significantly increased viability when compared to the vehicle group (*n* = 6, *P* < 0.01). Similarly, PI/Hoechst staining also showed higher cell death percentage at 16 h and 24 h after GD compared to the control group (84.5 ± 5.1% and 95.7 ± 3.2% versus 3.2% ± 1.1%, *n* = 6, *P* < 0.01), but treatment with 100 *μ*M NSC or APO significantly mitigated GD-induced cell death in photoreceptors compared to the vehicle group (4.6 ± 0.9% and 9.6 ± 0.5% versus 84.5 ± 5.1%, *n* = 6, *P* < 0.01) (Figure 4). These results indicate that NOX2 activation is a key molecular step in the cell death cascade; inhibiting NOX2 could block GD-induced cell death.

### 3.3. NOX2 Is an Upstream Signal in the Caspase-Dependent Cell Death Cascade

**Figure 5:**
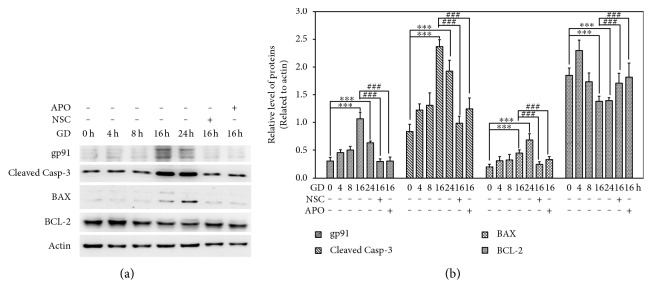
Inhibition of NOX2 suppresses caspase-dependent apoptotic pathway. (a) 661W cells were cultured in normal, glucose-free medium, or in the presence of 100 *μ*M NSC or APO for indicated time periods. Cell lysates were collected and subjected to Western blot analysis of gp91, cleaved caspase-3, Bax, and Bcl-2. Actin protein was used as a loading control. (b) Protein bands on the Western blots were scanned, and the intensity was determined by optical density measurements. The results are presented as means ± SEM (*n* = 3, ^∗∗∗^*P* < 0.01 compared to the control group, ^###^*P* < 0.01 compared to the vehicle group; one-way ANOVA and Bonferroni test).

**Figure 6:**
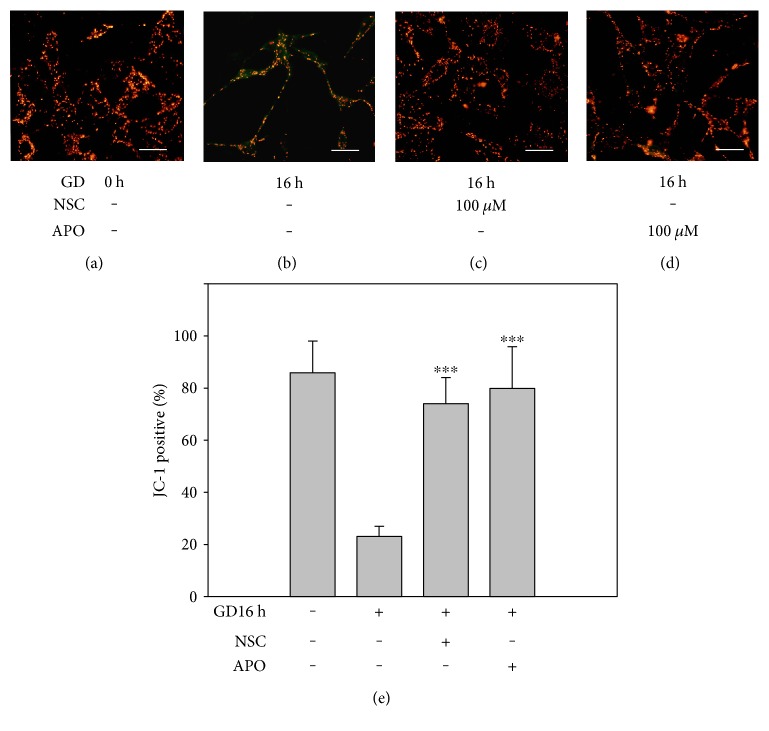
Inhibiting NOX2 improves the mitochondrial membrane potential. The mitochondrial membrane potential was analyzed by JC1 staining under a fluorescence microscope. The mitochondria with normal Δ*ψ*m were stained with red punctuated fluorescence (JC-1 positive), but mitochondria with depolarized Δ*ψ*m were stained with green fluorescence. (a) 661W cells were cultured in normal medium. (b) 661W cells were cultured in glucose-free medium for 16 h. (c), (d) 661W cells were cultured in glucose-free medium for 16 h in the presence of 100 *μ*M NSC (c) or 100 *μ*M APO (d). Scale bar = 50 *μ*m. (e) The percentage of JC-1-positive cells was quantitatively assessed with flow cytometry. Assays were performed in quadruplicate, and data are shown as means ± SEM (^∗∗∗^*P* < 0.01 compared to the vehicle group; one-way ANOVA and Bonferroni test).

To better illustrate NOX2 activation, we measured the expression of gp91, the core catalytic subunit of NOX2, during the course of GD. As shown in Figure 5, GD led to a significant increase in gp91 after 16 h when compared to the control (*n* = 3, *P* < 0.01), while this upregulation was mitigated by treatment with 100 *μ*M APO or NSC (*n* = 3, *P* < 0.01). In addition, GD induced caspase-dependent cell death in photoreceptors as evidenced by a significant upregulation of cleaved caspase-3 after 16 h with Western blot (*n* = 3, *P* < 0.01). However, treatment with 100 *μ*M APO or NSC also reduced the amount of cleaved caspase-3 when compared to the vehicle group (*n* = 3, *P* < 0.01). To assess mitochondrial function, we determined mitochondrial outer membrane permeabilization (MOMP) formation related preapoptotic factor (Bax), and proapoptotic factor (Bcl-2) and the mitochondrial membrane potential (Δ*ψ*m). After 16 h of GD, Bax was upregulated and Bcl-2 was accordingly downregulated compared to the control (*n* = 3, *P* < 0.01). However, treatment with NSC or APO significantly reversed these trends shown in Figure 5 (*n* = 3, *P* < 0.01). In addition, Figure 6 shows that Δ*ψ*m was depolarized compared to the control cells as evidenced by green fluorescence instead of reduced red fluorescence at 16 h after GD and decreased percentage of JC-1-positive cells compared with the control group (23 ± 5% versus 88 ± 14%, *n* = 6, *P* < 0.01). However, treatment with 100 *μ*M APO or NSC significantly attenuated changes in Δ*ψ*m, as indicated by increased red fluorescence and the percentage of JC-1-positive cells when compared to the vehicle group (74 ± 15% and 76 ± 21% versus 23 ± 5%, *n* = 6, *P* < 0.01). These results suggest that NOX2, an upstream signal, is involved in GD-induced caspase-dependent cell death.

### 3.4. The Downstream MAPK Signal Pathways Are Activated

**Figure 7:**
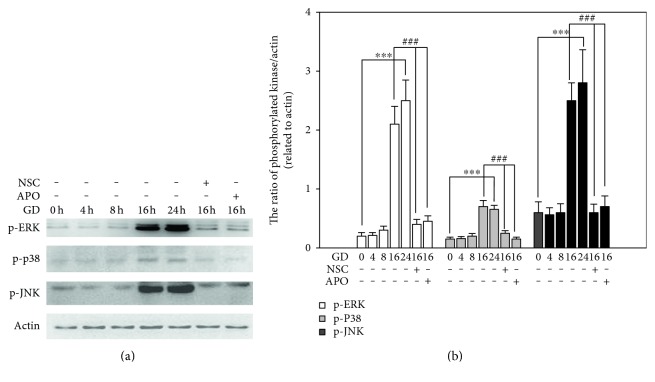
Inhibition of NOX2 suppresses the activation of MAPK signals. (a) 661W cells were cultured in normal or glucose-free medium and cells were treated with 100 *μ*M NSC or APO for 16 h. Cell lysates were collected at the indicated time periods and subjected to Western blot analysis of phosphorylated ERK, JNK, and p38. Actin protein was used as an internal control. (b) Protein bands on the Western blots were scanned, and the intensity was determined by optical density measurements. The ratio of phosphorylated kinase/*β*-actin was calculated based on the values of optical density. The results are presented as means ± SEM (*n* = 3, ^∗∗∗^*P* < 0.01 compared to the control group, ^###^*P* < 0.01 compared to the vehicle group; one-way ANOVA and Bonferroni test).

MAPK signaling cascades involve highly conserved serine/threonine kinases that connect cell surface receptors to regulatory targets in response to oxidative stress. We assessed the activation of MAPK signal in GD-induced cell death by measuring three kinase subfamilies: JNK, p38, and ERK. 661W cells were cultured in glucose-free medium for various time periods (0–24 h) and the active forms p-ERK, p-JNK, and p-P38 were determined by Western blot analysis. As shown in Figure 7, GD caused remarkable upregulation in the levels of both phosphorylated ERK, JNK, and P38 at 16 h and 24 h. Further quantitative analysis showed that the ratios of p-ERK/*β*-actin, p-JNK/*β*-actin, and p-P38/*β*-actin were significantly increased at 16 h and 24 h when compared to the control groups (*n* = 3, *P* < 0.01). Next, we monitored the effect of NOX2 inhibition on MAPK signals. As shown in Figure 7, treatment with 100 *μ*M NSC and APO at 16 h after GD remarkably reduced both p-ERK, p-JNK, and p-P38 levels, and the ratios of phosphorylated kinases over *β*-actin significantly decreased as well (*n* = 3, *P* < 0.01). These results indicated that GD caused the activation of MAPK signals, yet NOX2 functioned as the upstream signal that regulates the MAPK signal.

### 3.5. MAPK Signals Are Involved in the GD-Induced Cell Death Cascade

**Figure 8:**
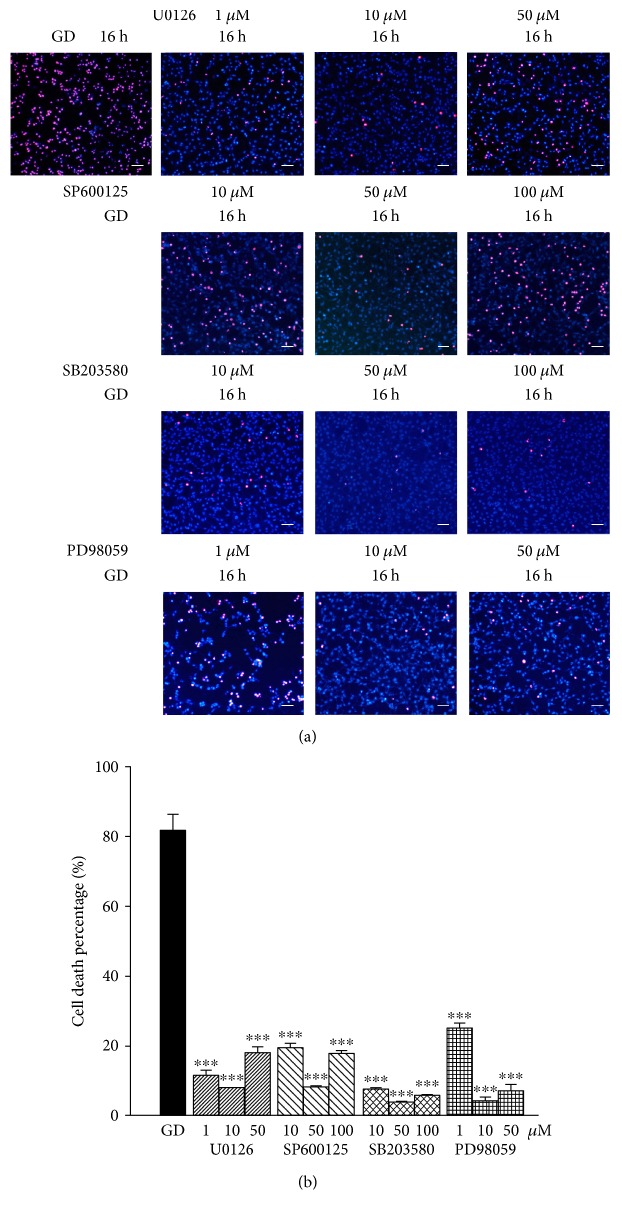
Inhibiting MAPK signal protects 661W cells from GD-induced death. Cell death was evaluated with PI/Hoechst staining. PI-positive cells were identified with purple fluorescence, and total cells were counter stained with Hoechst showing blue fluorescent nuclei. (a) 661W cells were cultured in glucose-free medium for 16 h as a vehicle control. 661W cells were treated with U0126 (1–50 *μ*M), SP600125 (10-100 *μ*M), SB203580 (10-100 *μ*M), and PD98059 (1–50 *μ*M) during GD. Scale bar = 200 *μ*m. (b) Quantitative assessment of cell death was performed through PI/Hoechst staining. Scale bar = 200 *μ*m. Assays were performed in quadruplicate, and data are shown as means ± SEM (^∗∗∗^*P* < 0.01 compared to the vehicle group; one-way ANOVA and Bonferroni test).

To further investigate the role of MAPK signals, we monitored the effects of their inhibitors on GD-induced cell death. As shown in Figure 8, application of the specific inhibitors, PD98059, U0126 (MEK/ERK inhibitor, 1–50 *μ*M), SP600125 (JNK inhibitor, 10–100 *μ*M), and SB203580 (P38 inhibitor, 10–100 *μ*M) significantly reduced GD-induced cell death at 16 h evidenced by remarkably lesser number of cells that stained positive for PI compared to the vehicle group (*n* = 6, *P* < 0.01). U0126 and PD98059 at 10 *μ*M were the most protective (8.0 ± 0.3%, 5.2 ± 1.1% versus 83.5 ± 8.3%, *n* = 6, *P* < 0.01), whereas SP600125 and SB203580 at 50 *μ*M showed the best neuroprotection (8.1 ± 0.5%, 5.0 ± 0.5% versus 83.5 ± 8.3%, *n* = 6, *P* < 0.01). These results indicate that MAPK signals are a key step in the cell death molecular mechanism and blocking MAPK signals could effectively protect 661W cells from acute energy depletion-induced cell death.

### 3.6. NF-*κ*B Signal Is Activated and Involved in GD-Induced Death

**Figure 9:**
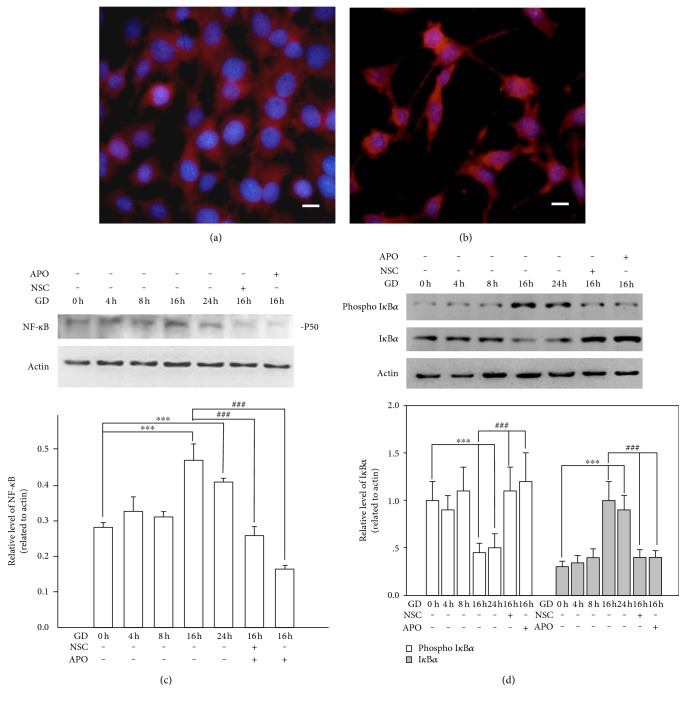
Inhibition of NOX2 suppresses the activation of NF-*κ*B signal. NF-*κ*B expression was specifically identified with immunohistochemistry assay and nucleus was counter stained with Hoechst. (a) The cells cultured in normal medium showed that NF-*κ*B (p65) was mainly expressed in the cytosol, which clearly showed the Hoechst stained blue nucleus. (b) The cells cultured in glucose-free medium for 16 h and the translocation of NF-*κ*B (p65) from cytosol to nucleus was identified by the purple-colored nucleus. Scale bar = 25 *μ*m. (c), (d) 661W cells were cultured in normal or glucose-free medium and cells were treated with 100 *μ*M NSC or APO for 16 h. Cell lysates were collected at the indicated time periods and subjected to Western blot analysis of NF-*κ*B (p50), phosphorylated IkB*α*, and nonphosphorylated IkB*α*. Actin protein was used as a loading control. Protein bands on the Western blots were scanned, and the intensity was determined by optical density measurements. The results are presented as means ± SEM (*n* = 3, ^∗∗∗^*P* < 0.01 compared to the control group, ^###^*P* < 0.01 compared to the vehicle group; one-way ANOVA and Bonferroni test).

**Figure 10:**
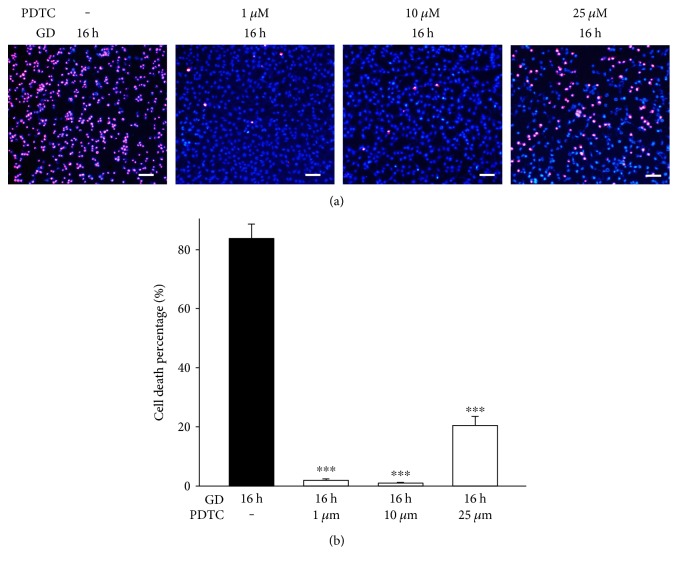
Inhibiting the NF-*κ*B signal protects 661W cells from GD-induced cell death. Cell death was evaluated with PI/Hoechst staining. PI-positive cells were identified with purple fluorescence and total cells were counter stained with Hoechst showing blue fluorescent nuclei. (a) 661W cells were cultured in glucose-free medium for 16 h as a vehicle control. 661W cells were treated with (1–25 *μ*M) PDTC during the course of GD. Scale bar = 200 *μ*m. (b) Quantitative assessment of cell death was performed through PI/Hoechst staining. Scale bar = 200 *μ*m. Assays were performed in quadruplicate, and data are shown as means ± SEM (^∗∗∗^*P* < 0.001 one-way ANOVA and Bonferroni test, compared to the vehicle control).

Since NF-*κ*B is another important signal pathway sensitizing intracellular oxidative stress, we assessed its role in GD-induced cell death. During activation, NF-*κ*B translocates from the cytosol into nucleus where it regulates transcription of downstream genes; therefore, we traced NF-*κ*B translocation by immunohistochemistry. As shown in Figure 9(a), NF-*κ*B (p65) is mainly expressed in the cytosol in normal cultured cells, which clearly stained blue nuclei with Hoechst. However, NF-*κ*B (p65) is apparently translocated into the nucleus as indicated by the purple-colored nuclei at 16 h after GD treatment (Figure 9(b)). Next, we measured NF-*κ*B expression using Western blot analysis.  Figure 9(c) shows that GD markedly activated NF-*κ*B (P50 subunit) at 16 h and 24 h (*n* = 3, *P* < 0.01), while treatment with 100 *μ*M NSC and APO significantly attenuated the activation after 16 h of GD treatment (*n* = 3, *P* < 0.01). Moreover, the level of I*κ*Ba, the inhibitor protein of NF-*κ*B, significantly decreased during GD, whereas the level of phosphorylated I*κ*Ba increased consistently. Yet, treatment with 100 *μ*M NSC and APO markedly reversed this process (Figure 9(d)). Additionally, blockage of NF-*κ*B signal with its specific inhibitor, PDCT (1 *μ*M–25 *μ*M), remarkably rescued photoreceptors from GD-induced cell death at 16 h, which significantly reduced cell death rate (71% ± 5% versus <20% ± 5%, *n* = 6, *P* < 0.01) (Figure 10). These results indicate that NF-*κ*B also plays an important role in the cell death cascade.

## 4. Discussion

Glucose deprivation results in an imbalance between prooxidant factors and antioxidant factors, such as reduced glutathione levels and increased expression of GSH peroxidase, which cause severe oxidative stress [11]. Our results demonstrated that photoreceptors underwent severe oxidative stress at 16 h after GD, which produced massive amounts of intracellular ROS detected by the DHE assay. This action was mediated via upregulating endogenous antioxidant defense components including HO-1. HO-1 is induced by its substrate heme, as well as by various oxidative stresses [12] and is thought to play an important protective role against oxidative injuries [13]. We detected that the level of HO-1 in photoreceptors was significantly increased after 16 h GD and lasted until 24 h. The GD-induced oxidative stress could be the initial signal to trigger the activation of the cell death cascade. In most cases, cells undergo apoptosis via engagement of the intrinsic pathway, the regulation of which is centered in the mitochondria. The localization and control of Bcl-2 proteins on mitochondria is essential for the intrinsic pathway of apoptosis [14]. The Bcl-2 family proteins are the essential regulators of MOMP, and the correct targeting of the Bcl-2 family to mitochondria is critical for apoptosis [15]. Antiapoptotic Bcl-2 proteins reside on the outer mitochondrial membrane (OMM) and prevent apoptosis by inhibiting the activation of the proapoptotic family members, Bax and Bak. The multidomain proapoptotic proteins, Bax and Bak, are thought to promote MOMP by oligomerizing to form pores within the OMM [16]. Our results demonstrated that the antiapoptotic factor, Bcl-2, was apparently downregulated after 16 h in GD-induced cell death, yet, the proapoptotic protein, Bax, was increased as detected in Western blots. The dysfunction of mitochondria was also demonstrated by measuring mitochondrial membrane potential. The massive cells lost functional Δ*ψ*m after 16 h of GD, showing increased green fluorescence instead of red fluorescence by JC-1 staining. The formation of MOMP and clapsion of mitochondrial membrane potential resulted in the release of soluble factors from the intermembrane space. The key factors released, cytochrome c and SMAC/DIABLO (second mitochondria-derived activator of caspase/direct inhibitor of apoptosis-binding protein with low pI), interact with cytosolic factors to activate the caspases. As a result, we detected that the cell death executor, caspase-3, was activated and that the cleaved form was upregulated after 16 h of GD. Since some cells die even in normal culture conditions, the cleaved caspase 3 could also be detected in the control group.

NOXes are an important source to generate intracellular ROS, but neurons mainly express the NOX2 isoform. When NOX2 is activated, its cytosolic subunits bind two membrane-bound subunits, p22phox and gp91phox (the catalytic unit), and the small GTPase Rac1 to form an active transmembrane enzyme complex [5]. In this study, we demonstrated that GD can activate NOX2 because gp91 was significantly upregulated at 16 h and 24 h of GD treatment. Our results also revealed that NOX2 is a dominant source that caused GD-induced superoxide generation since inhibiting NOX2 with APO [17–19] reduced gp91 expression and blocked superoxide formation and photoreceptor cell death. Rac1 is an additional requisite component of functional NOX2 oxidase and a Rho GTPase signaling protein and functions as a guanine nucleotide exchange factor to catalyze the conversion of inactive GDP-bound GTPases to active GTP-bound complexes. To further illustrate the role of NOX2 in cell death, we suppressed the activity of RAC1 with its inhibitor. We found that the Rac inhibitor, NSC, attenuated NOX2 activation and reduced oxidative stress and neuronal death [20]. We used two methods to assess the neuroprotective effect of NOX2 inhibition, PI/Hoechst staining and MTT assay. PI is a fluorescent molecule, is membrane impermeable, and is excluded from viable cells. Therefore, PI is commonly used for identifying dead cells in a population. Normally, apoptotic cells are characterized by DNA fragmentation and, consequently, loss of nuclear DNA content. Use of a fluorochrome, such as PI, that is capable of binding and labeling DNA makes it possible to obtain a rapid and precise evaluation of apoptotic cells [21]. However, MTT assay is a colorimetric assay for assessing cell metabolic activity. NAD(P)H-dependent cellular oxidoreductase enzymes are capable of reducing the tetrazolium dye MTT (3-(4,5-dimethylthiazol-2-yl)-2,5-diphenyltetrazolium bromide) to its insoluble form called formazan, which has a purple color. Therefore, reduction of MTT and other tetrazolium dyes depends on the cellular metabolic activity due to NAD(P)H flux. Cells with low metabolism reduce very little MTT. In contrast, rapidly dividing cells exhibit high rates of MTT reduction [22]. Because PI staining and MTT assay use different mechanisms to evaluate dead cells and cell viability, the absolute value of data we obtained in assessing the neuroprotective action of NOX2 inhibition is a little different, but the final tendency is quite consistent. To better illustrate whether NOX2 is the upstream signal of GD-induced cell death, we measured its effects on downstream factors. We showed that inhibition of NOX2 with APO or NSC reduced the active form of caspase-3, downregulated BAX, and upregulated Bcl-2. From JC-1 staining, we also observed that inhibition of NOX2 maintained integrity of mitochondrial outer membrane and Δ*ψ*m during glucose starvation. Therefore, these results provide evidences that NOX2 may be the primary source to generate ROS, which may function as an upstream signal in the death cascade, and suppression of oxidative stress is a key point in rescuing photoreceptors from GD-induced cell death.

Three subgroups of MAPKs are involved in both cell growth and cell death [23]. ROS such as hydrogen peroxide have been reported to activate ERKs, JNKs, and p38 MAPKs. Our results show that all three subpathways were activated during GD. The ratios of p-ERK/*β*-actin, p-JNK/*β*-actin, and p-P38/*β*-actin significantly increased at 16 h and 24 h of GD treatment and yet treatment with NOX2 or RAC1 inhibitors remarkably reduced the increase in these ratios. ROS derived from NOXes can specifically and reversibly react with proteins, rapidly oxidizing the highly reactive thiol groups to form disulfide bonds [24]. The oxidized target proteins activate a number of oxidation-sensitive processes that bring about a number of cellular responses, such as gene activation, modulation of ion channels, and the activity of other signaling pathways, including MAPK cascade. Among the MAP3K family, ASK1 (apoptosis signal-regulating kinase 1) has been extensively characterized as an ROS-responsive kinase, which possesses a serine/threonine kinase domain in the middle part of the molecule flanked by the N- and C-terminal coiled-coil (CCC) domains. ROS is the most potent activator of ASK1 [25]. In ASK1-deficient cells, ROS activated JNK in a consistent manner [26], suggesting that ASK1 is one of the upstream kinases that can sense the ROS signal and translate it to downstream kinases [27]. In this study, we found that blocking ERK, P38, and JNK pathways significantly attenuated GD-induced cell death, which suggests that MAPK signals are involved in the apoptotic cascade. MAPKs are serine/threonine kinases that, upon stimulation, phosphorylate their specific substrates at serine and/or threonine residues. Such phosphorylation events can either positively or negatively regulate substrate and thus the entire signaling cascade activity. Thus, the MAPK signaling pathways modulate gene expression, proliferation, motility, metabolism, and programmed cell death [28–34].

NF-*κ*B proteins are a family of transcription factors that regulate expression of numerous genes. There are five known family members binding as homodimers or heterodimers to 10 base pair *κ*B sites. These are subunits p50 (derived from p105), p52 (derived from p100), p65 (RelA), c-Rel, and RelB. These subunits form biologically active molecules of NF-*κ*B, which translocate to the nucleus upon phosphorylation and transcribe various genes [35]. GD caused a massive increase in intracellular ROS generation. Several lines of evidence suggest that ROS mediates the activation of redox-sensitive transcription factors such as NF-*κ*B. However, how exactly ROS activates various protein kinases upstream to NF-*κ*B is not clear. Evidence suggests that ROS induced lipid peroxidation products such as lipid aldehydes during the activation of signaling cascade that eventually activate NF-*κ*B [21, 36–39]. In this study, we found that NF-*κ*B was activated after 16 h of GD along with upregulation of P50 subunit and translocation of NF-*κ*B (p65) from cytosol to nucleus. Traditionally, NF-*κ*B is sequestered in the cytoplasm by its inhibitor protein, I*κ*Ba [40]. NF-*κ*B activation can induce I*κ*B phosphorylation, which releases NF-*κ*B to the nucleus, where it binds to specific sequences in the promoter regions of genes [41]. We also observed that the phosphorylated I*κ*Ba significantly increased during GD, especially at 16 h and 24 h, whereas nonphosphorylated I*κ*Ba decreased consistently. Interestingly, both antiapoptotic and proapoptotic effects of NF-*κ*B have been reported previously. Activation of NF-*κ*B was neuroprotective for *β*-amyloid-triggered cell death [42]. However, *β*-amyloid 40 activated the nuclear translocation of p50/p65 dimers, which promoted a proapoptotic profile of gene expression and inhibitors of the NF-*κ*B pathway specifically targeting p50/p65 dimers could be considered for blocking neurodegeneration [43]. In this study, we found that NF-*κ*B plays a proapoptotic role in GD-induced cell death since treatment with the NF-*κ*B inhibitor significantly rescued photoreceptors from death. Yet, the influence of NF-*κ*B signal on the cell death cascade and the crosstalk between MAPK and NF-*κ*B signaling pathways in this cell model should be demonstrated in further studies.

In summary, acute energy depletion induced caspase-dependent cell death, but NOX2 as its upstream signal regulated the cell death cascade. Activation of NOX2 produced massive ROS, which further caused the activation of the MAPK and NF-*κ*B signals. Blockage of either MAPK family members or NF-*κ*B could effectively rescue photoreceptors from GD-induced cell death. These findings may provide insights to treat ischemic-related retinal diseases.
